# Editorial: Advances in precision livestock management for grazing ruminant systems

**DOI:** 10.3389/fvets.2026.1851831

**Published:** 2026-05-04

**Authors:** Paulo de Mello Tavares Lima, Tiago do Prado Paim

**Affiliations:** 1 Department of Animal Science, University of Wyoming, Laramie, WY, United States; 2 Embrapa Pesca e Aquicultura, Palmas, TO, Brazil

**Keywords:** animal grazing behavior, animal health, application of novel technology, feed intake, methane measurement, remote sensing, RFID, virtual fencing

Precision livestock management (PLM) refers to a set of practices and tools designed to enable precise and continuous monitoring of animal health, welfare, productive performance, reproductive efficiency, and the environmental impacts within livestock production systems (1, 2). Achieving this level of monitoring can be particularly challenging in grazing ruminant systems, which are typically extensive in spatial scale and characterized by significant resource heterogeneity. Variability in elevation, soils, plant communities, and topography—combined with dynamic spatiotemporal climatic conditions—creates environments that are difficult, if not impossible, for humans to manage for livestock production and other ecosystem services (3–5). Recent technological advances have lowered costs and improved access for both producers and researchers to PLM sensors, electronic meters, and other tools. These innovations enable the detection and monitoring of animal movement, physiological and reproductive status, grazing behavior, health conditions, rumination patterns, feed intake, welfare conditions, performance, and even greenhouse gas emissions (2, 5). This Research Topic brings together 11 studies (Table 1) that explore the application of precision livestock management technologies in grazing systems.

**Table 1:** Summary of manuscripts publish on the Research Topic—*Advances in precision livestock management for grazing ruminant systems*.

Published manuscripts	PLM tool and/or subject
Zwygart et al.	Animal welfare
Kersh et al.	Metabar coding for feed selection in grazing sheep
Qin et al.	Animal identification
Fu et al.	Molecular biology
Tong et al.	Animal welfare
Vandermark et al.	GPS, virtual fencing, in-pasture weighing system
McFadden et al.	Methane emissions, feed intake, body weight measurement
Morales-Vargas et al.	Internet of things, imagery, and animal behavior.
Raynor et al.	Virtual fencing
Irisarri et al.	Remote sensing and feed quality
Han et al.	Animal identification

PLM, precision livestock management.

A study on livestock welfare was developed by Zwygart et al.. The authors evaluated the feasibility of assessing welfare in Swiss veal production using routinely collected digital data. While indicators such as morbidity, mortality, and body condition were available, they mainly reflected animal health rather than welfare, and key behavioral data were lacking. Additionally, much of the data originated from slaughterhouses but not farms, therefore limiting its usefulness for producers. These gaps highlight the potential of PLM technologies to improve on-farm welfare assessment. Tong et al. developed an automated system to assess animal welfare by integrating animal behavior, environmental conditions, and feeding management in feedlot dairy cattle. The model combined fuzzy logic with parallel backpropagation neural networks using Gaussian membership functions. It showed excellent performance and potential for broader application in modern farm management.

Kersh et al. implemented fecal DNA (f.DNA) metabarcoding technology in tandem with focal bite count observations to estimate diet composition of sheep during targeted grazing in a northern mixed-grass prairie. Fecal DNA technology was effective at establishing relative and semi-quantitative comparisons among individual sheep and examining temporal trends in diet selection. Together, insights from focal bite count observations and f.DNA could be used to support producer decisions within an adaptive management framework. Expansion of diet estimation technologies could improve adaptive decision making within broader PLM applications in extensively managed systems.

Facial color patterns are a key criterion in breeding decisions for Ujumqin sheep, a fat-tailed breed from Inner Mongolia, China. Animals with a white head and five black points are preferred for reproduction. Qin et al. used the YOLOv8 image detection model, enhanced with a Convolutional Block Attention Module (CBAM), to automate classification of these patterns. This PLM approach improved accuracy and efficiency in selection for producers. The authors suggest that future work should focus on improving performance under varying lighting conditions and shooting distances to increase model robustness. Also in China, Fu et al. studied the local breed Honghe cattle to evaluate the effects of feedlot vs. grazing systems on rumen health. Although not using typical PLM tools, they found that feedlot animals showed improved rumen barrier function and antioxidant status. While diet plays a major role in such results, the study highlights the potential of molecular biology to inform decision-making in animal production systems.

Based on nutritional models, Vandermark et al. improved estimates of energy requirements in grazing beef cattle by combining GPS collars with in-pasture weighing systems under continuous and rotational grazing managed via virtual fencing (VF). Conducted in the rangelands of the intermountain west region of the U.S.A., a heterogeneous landscape, the study showed that topography, grazing system (i.e., continuous vs. rotational), and stocking rate influence energy demands. These findings called attention to the potential of PLM tools to enhance nutritional efficiency in grazing systems. On another U.S. rangeland study, McFadden et al. used a suite of PLM technologies (GreenFeed™, SmartFeeder™, and SmartScale™—C-Lock, Rapid City, SD, USA) to measure gas emissions, oxygen consumption, dry matter intake (DMI), and body weight in grazing beef cattle. They developed a model to predict DMI, with the best performance obtained using smoothed herd data (*R*^2^ = 0.77). Results provide a starting baseline for estimating intake from enteric gas emissions in grazing systems and should guide future research on this area.

In a study of grazing dairy cows in southern Chile, Morales-Vargas et al. used Internet of Things (IoT) collars and pasture cameras to collect GPS, accelerometer, and behavioral data. Machine learning algorithms classified behaviors such as walking, grazing, and resting to support early lameness detection. Although based on a small sample, the publicly available dataset provides a useful foundation for developing reliable lameness monitoring systems in grazing conditions, with potential benefits for productivity and animal welfare.

Raynor et al. evaluated animal performance and CH_4_ emissions in yearling steers using a VF system in shortgrass prairie. The goals of this study were to assess the efficiency of the VF in establishing a rotational grazing system and to estimate the impacts of this PLM tool on emissions and animal weight gain. The system effectively controlled grazing, with animals respecting boundaries 94 and 99% of the time. However, VF management did not improve weight gain or consistently reduce emissions, likely due to factors such as forage quality. The authors emphasized the need to incorporate forage quality into future studies to optimize grazing strategies.

In a study conducted in southwest UK, Irisarri et al. aimed to improve the estimation of forage quality in pasture landscapes. The authors assessed key forage quality attributes—crude protein, water-soluble carbohydrates, neutral detergent fiber, and acid detergent fiber—using near-infrared spectroscopy (NIRS) and compared these measurements with estimates derived from Sentinel-2 remote sensing imagery. The results showed strong agreement between the two approaches, with *R*^2^ values ranging from 0.77 to 0.86 and low root mean square error, indicating high model accuracy and highlighting its potential for large-scale forage quality monitoring.

Focusing on animal identification tools, Han et al. improved cattle identification using muzzle images by developing a Siamese neural network trained on 31,312 images from 658 animals under varied, real-world conditions. The model achieved 97.9% accuracy, demonstrating strong potential for applications such as agricultural insurance verification and improved efficiency in livestock management systems.

This Research Topic represents a highly valuable compilation of cutting-edge PLM tools aimed at improving the management of grazing livestock under diverse conditions worldwide. Although many of these technologies are still in the early stages of development and application, further refinement and a deeper understanding of their full potential are expected in the coming years. Nevertheless, the contributions gathered here provide an important overview of currently available technologies and demonstrate how they can be leveraged to enhance the efficiency and sustainability of livestock production systems.
